# The Influence of Face on Online Purchases: Evidence From China

**DOI:** 10.3389/fpsyg.2021.788063

**Published:** 2021-11-03

**Authors:** Gong Sun, Famei Shen, Xinyi Ma

**Affiliations:** ^1^Business School, Changshu Institute of Technology, Changshu, China; ^2^International Business School Suzhou, Xi’an Jiaotong-Liverpool University, Suzhou, China; ^3^Business School, Central University of Finance and Economics, Beijing, China

**Keywords:** face, online purchases, materialism, price consciousness, China

## Abstract

This study examines the influence of an indigenous concept – face on online purchases in China. Specifically, we test the mediating role of materialism and the moderating role of price consciousness. We conduct a survey of 315 Chinese consumers. The results demonstrate that consumers tend to make online purchases to satisfy some facets of their materialistic needs and then gain face, especially for those highly conscious of price. The current research can help both scholars and practitioners better understand Chinese consumers and their decision-making processes. Implications, limitations, and directions for future research are also provided.

## Introduction

Over the last two decades, the Internet has developed considerably in China. As of December 2020, the number of netizens in China reached 989 million, accounting for approximately one-fifth of the world’s netizens, among which the number of online shopping users reached 782 million, accounting for 79.1% of the total number of netizens (China Internet Centre, 2021). The development of the Internet in China has also promoted the rapid growth of the e-commerce industry. The 2019 China E-commerce Report shows that the transaction volume of China’s e-commerce market in 2019 reached 34.81 trillion RMB with a year-on-year growth level of 6.7%, of which online retail sales reached 10.03 trillion RMB with a year-on-year growth level of 16.5% (Global E-Commerce Sumit, 2020).

Due to its profound influence on human behavior, culture has been widely considered in consumer research (Craig and Douglas, 2006; Yaprak, 2008). However, most cultural concepts applied in current consumer studies are derived from Western societies (Sun et al., 2014b). Such approaches might not be able to measure the nuances of Eastern societies such as China and then may fail to accurately illustrate business phenomena in such societies (Adler et al., 1989; Redding, 1990). Even though Western culture has become increasingly pervasive in recent decades, China has maintained its traditional values and ways of thinking to a great extent (Ackerman et al., 2009). Such concepts are based on social standards and norms that have been embedded in the country for thousands of years and that still regulate people’s daily lives (Yau, 1988; Yau et al., 1999). Therefore, it is meaningful to explore roles of such concepts in consumers’ decision-making processes regarding online purchases.

One of the most significant Chinese cultural orientations is face (*mianzi*), which is defined as the sense of favorable social self-worth that a person wants others to have of him or her in social interactions (Goffman, 1967). Face reflects one’s social image or self-esteem, and most people tend to make efforts to gain respect from others in relational contexts (Ting-Toomey and Kurogi, 1998). In Western societies, people are generally high in individualism and more likely to make decisions based on their own inclinations and preferences (Markus and Kitayama, 1991; Triandis, 1995). However, in China, which is an extremely collectivistic country, levels of social normative influence are quite strong, and behavioral deviance is not as acceptable as it is in Western countries (Gelfand et al., 2006). Thus, if Chinese individuals’ behaviors fail to meet the expectations of others, they may lose face (Zhang et al., 2011). Therefore, Chinese consumers tend to buy specific products that signal certain meanings to gain face or avoid losing face (Sun et al., 2015; Wang et al., 2019). Sometimes, such individuals do not “want to” but “have to” make these purchases (Wong and Ahuvia, 1998). Online shopping affords consumers greater degrees of convenience and accessibility. Consumers can obtain access to online shops wherever they are. They can make full use of their fragmented time. Online shopping also benefits individuals who do not like interacting with sales assistants (He et al., 2018), thus encouraging them to buy products that can enhance their social image. In another respect, He et al. (2018) find that a major motivation for online shopping among Chinese consumers is to engage in “observed buying” rather than “unobserved shopping,” which is found in Western culture. The authors explain that this is the case because consumers may choose to receive their purchases at their office or school, which can help them gain face in the presence of others such as colleagues and classmates. Therefore, we hope to explore the role of face in online shopping behavior in China.

He et al. (2018) found that compulsive buying, defined as a tendency to be preoccupied with compulsive purchasing, relates to both face and online buying motivations in China. However, compulsive buying tendencies mainly satisfy consumers’ self-directed emotional and hedonic needs. Consumers may purchase products for socially focused needs, such as to signal status and wealth, rather than to merely obtain instant gratification. Therefore, we believe that compulsive buying is not comprehensive in predicting online shopping behavior in China. He et al. (2018) attributed the prevalence of compulsive buying to the spread of materialistic culture in China since the opening up policy of 1978. Materialism has become the main social and economic ideology in today’s China and has effects on consumption behaviors (Sun et al., 2014a; Yang and Stening, 2016). Previous studies have conceptually and empirically explored the relations between face and materialism. Hence, we posit that materialism may relate face consciousness to online buying behavior.

In addition, although materialistic values are already rooted in today’s society, Chinese consumers are still very sensitive to price (Faure and Fang, 2008). Resource scarcity since ancient times has cultivated a tradition of thriftiness in China (Wang and Lin, 2009). Even though the country has experienced substantial economic growth in recent decades and is ranked second in economic scale worldwide, China is still a developing country. China’s GDP per capita was only approximately one-fifth of that of the United States and one-third of that of Japan. Therefore, most Chinese consumers are still price conscious. Zhan and He (2012) found that Chinese consumers focus on price even when they purchase luxury items. In China, due to its developed logistics system, low labor costs and high real estate rents, online sellers offer major cost advantages over traditional offline shopping channels. For the same products, online prices are much lower than that those of offline retailers. Online shopping can greatly attract consumers, especially those who are highly price conscious. Hence, a moderating effect of price consciousness on the relationship between materialism and online shopping is expected.

## Face, Materialism, and Online Shopping

Since materialism has become the driving force behind modern consumer society, it has been studied in a broad range of disciplines, such as demography (Easterlin and Crimmins, 1991), political science (Inglehart, 1990), and social psychology (Kasser and Ryan, 1993; Kasser and Ahuvia, 2002). Many marketing scholars have posed their own definitions of materialism, but there are underlying similarities. Belk (1985, p. 291) defines materialism as “the importance a consumer attaches to worldly possessions.” Rassuli and Hollander (1986, p. 10) view the term as “… an interest in getting and spending.” Browne and Kaldenberg (1997) identify materialism as a set of values that focus on possessions. In essence, these definitions relate to an individual’s emphasis on material possessions.

As the most widely used conceptualization of materialism, Richins and Dawson (1992, p. 307) propose that it “represents a mindset or constellation of attitudes regarding the relative importance of acquisition and possession of objects in one’s life.” The authors further operationalize materialism as a value that includes the dimensions of acquisition centrality, acquisition as the pursuit of happiness, and possession-defined success. Acquisition centrality proposes that materialists tend to place possessions and acquisition at the center of their lives. The pursuit of possessions structures their lives and directs their behavior. The second dimension is the extent to which materialists view possessions as essential to their life satisfaction and well-being. Hence, materialists pursue happiness through acquisition rather than through other means. Possession-defined success means that materialists judge their own and others’ success by the quality and quantity of their possessions.

The concept of face originating from Chinese culture represents one’s prestige and status and has been a fundamental principle in Chinese society (Redding and Ng, 1982; Hwang, 1987). The term stresses individuals’ social needs and represents the social self (Liao and Wang, 2009). In recent years, scholars have demonstrated the significance of face in affecting consumer behavior. A great number of studies find the influence of face on status and conspicuous consumption (e.g., Sun et al., 2014a, 2015; Li et al., 2015). The results suggest that consumers in Eastern societies tend to make specific purchases to demonstrate their social image and status (Sun et al., 2016, 2017, 2021).

Because face describes one’s wealth and prestige (Hwang, 1987; Zhang and Cao, 2010), people with strong face consciousness tend to pursue money and material wealth, regardless of how rich or poor they are, to enhance their reputation and social status (Wong and Ahuvia, 1998). Hence, face heightens the features of materialism. For instance, Liao and Wang (2009) empirically explore how face mediates the relationship between materialism and brand consciousness, which refers to the mental orientation to purchase brand name goods.

In addition, online shopping provides consumers with considerable accessibility, extreme convenience and relative affordability for their purchases. Online shopping satisfies materialists’ needs for acquiring and possessing as many material goods as possible and then further improves their social image. Materialists who view acquisition and occupation as at the center of their lives are likely to engage in online shopping to achieve both instant gratification and long-term conspicuous values that signify their success and status. Moreover, He et al. (2018) propose a significant “observed buying” effect in Chinese online shopping. Specifically, consumers may obtain face when they receive the products they purchase online in the presence of peers. This effect is more obvious for those with high degrees of materialism because they are more likely to relate material possessions to success.

**H1:** Face positively influences online purchase.
**H2:** The relationship between face and online purchase is mediated by materialism.

## Price Consciousness, Materialism, and Online Shopping

Price consciousness refers to consumers’ focus on price and obtaining value for money when making purchasing decisions (Sproles and Kendall, 1986; Lichtenstain et al., 1993). Marketing scholars have explored the impact of price consciousness on various consumer behaviors. For instance, Palazon and Delgado (2009) found that price consciousness can affect consumers’ evaluations of price discounts and premium promotions. Kukar-Kinney et al. (2007) examined a model of consumers’ responses to various types of price-matching characteristics with varying levels of price consciousness. It was also found that price consciousness moderates the influence of consumers’ assessments of a coalition loyalty program on their psychological reactance (Chang and Wong, 2018). Thriftiness is a pervasive traditional value in China (Chinese Culture and Connection., 1987). Even today, a large proportion of the Chinese population especially those who live in inland and rural areas, is not wealthy, so frugality is still identified as a virtue (Wang and Lin, 2009). It is discovered that price consciousness is an important motive when Chinese consumers make purchase decisions (Fan and Xiao, 1998; Tai, 2005; Zhou et al., 2010).

Since price consciousness is positively related to frugality (Shoham and Brencic, 2004), we believe that it is a prevalent consumer trait that can affect shopping behavior in the Chinese context. Consumers high in price consciousness often seek low prices in the marketplace and enjoy the process of searching and shopping for low prices (Alford and Biswas, 2002). They also tend to purchase private labels or store brands rather than national brands (Sinha and Batra, 1999; Ailawadi et al., 2001). E-commerce platforms such as Pinduoduo and Taobao in China provide astonishingly inexpensive generic products. Even for branded products, online prices are lower than those of offline retailers. Consequently, compared to less price conscious consumers, highly price conscious consumers are more likely to make online purchases to satisfy their material needs. Hence, a moderating role of price consciousness is proposed as follows.

**H3:** Price consciousness moderates the relationship between materialism and online purchases such that the relationship is stronger for those with higher rather than lower price consciousness.

**FIGURE 1:**
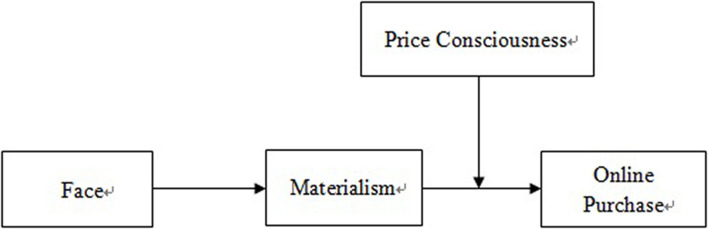
Hypothesized conceptual model.

Figure 1 shows the conceptual model of this study.

## Method

In the current study, snowball sampling through the use of social network sites was applied to collect data. We posted our questionnaires on Sojump,^1^ an online survey platform widely used for business and social research (Li et al., 2018). An attached cover letter assured participants that their participation was voluntary and anonymous and that their responses would be kept confidential. Finally, a total of 315 valid responses were included in this study. The participants included 133 males and 182 females, and more than 90% were between 21 and 30 years of age. Most of the respondents’ monthly incomes were less than RMB10,000.

## Measures

Most of the measures used are established scales. Face was measured by six items from Zhang et al.’s (2011) scale, which treats face consciousness as a general personality trait in social contexts. A sample item is “I hope people think that I can do better than most others.” In order to keep the survey at a reasonable length, materialism was measured using the six-item short form of the Material Values Scale (MVS) developed by Richins (2004), covering the three facets of materialism: possession-defined success, acquisition centrality and acquisition of happiness. Each facet includes two items. Sample items include “I admire people who own expensive homes, cars and clothes,” “Buying things gives me a lot of pleasure,” and “My life would be better if I owned certain things I don’t have.” We used three items from Sproles and Kendall (1986) to measure consumer price consciousness. A sample item is “the lower price products are usually my choice.” Moreover, we use an item, “How frequently you make online purchases in the last three months” to measure one’s online purchase behavior. Last, the study incorporates participants’ age, gender, and income as control variables. The translation and back-translation methods were applied to ensure that the questionnaire was easily understood by Chinese respondents (Brislin, 1970). All the constructs are measured with seven-point Likert scales.

## Results

This study tests the hypotheses with SPSS 22.0. In previous research, the three-facet structure of the materialism scale has not been cross-culturally validated, especially for non-English speaking countries (Griffin et al., 2004). Therefore, we first conducted an exploratory factor analysis (EFA) for the six items of materialism. The result shows that the dimensions of success and happiness merge as one factor and the dimension of acquisition centrality as the other factor. We use the two dimensions of materialism in the following analysis, which are denoted as materialism-S&H and materialism-AC, respectively. The Cronbach’s α values of the two dimensions are 0.746 and 0.632, respectively. Compared to previous relevant research (Sun et al., 2014a), the reliabilities of materialism in the present study show acceptable reliability. Face (α = 0.763) and price consciousness (α = 0.795) also achieve sound psychometric properties.

Then a confirmatory factor analysis (CFA) was performed with AMOS for all the latent variables to develop a good measurement model. The results of CFA indicated a sound model fit (Steiger, 1980): Chi-square = 134.9, df = 84, Chi-square/df = 1.61, CFI = 0.96, GFI = 0.95, TLI = 0.95, and RMSEA = 0.04. Moreover, all factor loadings were greater than the critical value of 0.5 (ranging from 0.52 to 0.80), showing adequate individual item reliability (Bagozzi and Yi, 1988). Table 1 shows the correlations between all the variables investigated in the current research. The result reflects that face positively correlates with materialism-S&H and online purchases, providing initial support for a mediation effect. Moreover, it is interesting that face has a positive correlation with price consciousness. It might be because both face and thriftiness are the core elements of Chinese traditional culture. In addition, materialism-AC negatively correlates with price consciousness, but positively correlates with online purchases.

**TABLE 1:** Correlations between the variables in the study.

	**1**	**2**	**3**	**4**	**5**	**6**	**7**	**8**
1. Face	–							
2. Materialism-S&H	0.52**	–						
3. Materialism-AC	–0.01	0.13*	–					
4. Price consciousness	0.14*	0.02	−0.26**	–				
5. Online purchases	0.27**	0.42**	0.13*	–0.09	–			
6. Age	–0.08	–0.01	−0.12*	–0.01	–0.03	–		
7. Gender	0.03	–0.02	0.15**	–0.03	0.01	−0.13*	–	
8. Income	0.02	0.08	0.02*	–0.07	0.14*	0.23**	−0.11*	–

*N = 315; gender: 1 = male and 2 = female.*

*p < 0.05; **p < 0.01.

To examine the mediating effect, the steps proposed by Baron and Kenny (1986) are adopted. First, the regression result shows that face is closely related to materialism-S&H (*r* = 0.515, *p* < 0.001) but does not significantly correlate with the acquisition centrality facet of materialism. The results shown in Table 2 indicate that after controlling for the demographic variables, the positive relationship between face and online consumption is significant (Model 2), which supports H1. When we include materialism-S&H in the regression (Model 3), the effect of face on online purchases disappears. The increased *R*^2^ value resulting from adding materialism-S&H to the equation is relatively large, demonstrating that the influence of face on online purchases is completely mediated by materialism-S&H. However, when we include materialism-AC in the regression model (Model 4), both face and acquisition centrality have impacts on online purchases.

**TABLE 2:** Results of the regression analyses.

**Variables**	**Online purchase**							
	**Model 1**	**Model 2**	**Model 3**	**Model 4**	**Model 5**	**Model 6**	**Model 7**	**Model 8**
**Control variables**								
Sex	0.017	0.011	0.022	–0.008	0.021	0.021	–0.001	–0.006
Age	–0.069	–0.047	–0.05	–0.032	–0.054	–0.075	–0.056	–0.055
Income	0.157**	0.146**	0.121*	0.138*	0.114*	0.126*	0.146*	0.146*
**Independent variable**								
Face		0.261***	0.069	0.264***				
**Mediator**								
Materialism-S&H			0.371***		0.410***	0.398***		
Materialism-AC				0.131*			0.111	0.113
**Moderator**								
Price consciousness					–0.089	–0.084	–0.050	–0.035
**Interaction term**								
Materialism-S&H × price consciousness						0.190***		
Materialism-AC × price consciousness								–0.052
*R* ^2^	0.024	0.092	0.192	0.108	0.196	0.232	0.041	0.044
Δ*R*^2^	0.024	0.067	0.1	0.017	0.172	0.036	0.017	0.002

*N = 315.*

*p < 0.05; **p < 0.01; ***p < 0.001.

We further examined the mediation effect of materialism-S&H between face and online purchase with the bootstrap method proposed by Hayes (2013). We set the random sampling size to 5,000 and the confidence interval to 95%. The results show that the indirect effect value of materialism is 0.269, and the 95% confidence interval is (0.180, 0.367), not including 0. It demonstrates that materialism fully mediates the influence of face on online purchase. Thus, H2 is partially supported for Materialism-S&H.

**FIGURE 2:**
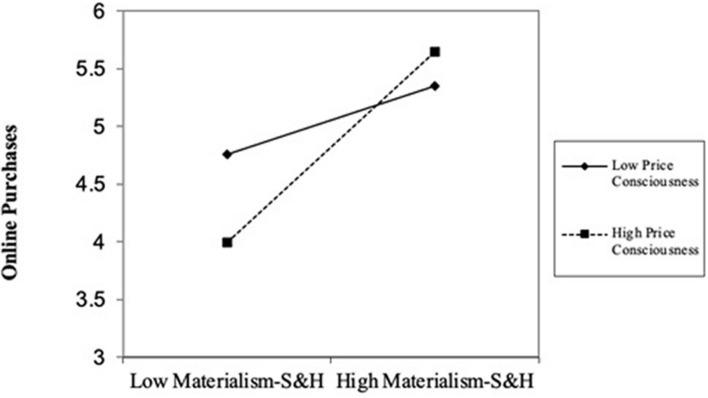
Interaction of materialism-S&H and price consciousness on online purchases.

Last, a hierarchical regression analysis is performed to test the moderating effect of price consciousness. Control variables, independent and moderating variables and the interaction term were added to the model in sequence. The results in Table 2 demonstrate that the interaction of materialism-S&H and price consciousness positively affects online purchases (*r* = 0.190, *p* < 0.001). The model achieves substantial incremental variance (Δ*R*^2^ = 0.036), and the change in *F* is significant at the *p* < 0.001 level. This result indicates that a higher level of price consciousness can lead to a stronger relationship between materialism-S&H and online purchases. The graphical depiction given in Figure 2 better illustrates the moderating effect of price consciousness. However, a moderating role of materialism-AC was not found.

## Discussion

The results of the current research explore the roles of face, materialism, and price consciousness in stimulating Chinese online shopping behavior. First, it is noteworthy that the three-factor structure of materialism originally derived from Richins and Dawson (1992) presents some unique features in the Chinese context. In this study, we extracted two factors from the six-item short-form MVS. Specifically, the dimensions of success and happiness are combined as one dimension, and acquisition centrality is used as another dimension. These two dimensions are weakly correlated, demonstrating that they are separate from each other. The intertwinement of success and happiness shows that Chinese consumers tend to acquire gratification by signifying their possession-defined success to others. This might be the case because in China and other Eastern countries where interpersonal influence is strong, people tend to endorse an interdependent sense-of-self that focuses on communal goals, norms, and obligations (Markus and Kitayama, 1991). Observing social norms and meeting others’ expectations constitute important sources of life satisfaction (Suh et al., 1998).

Acquisition centrality is a self-directed enduring value and thus not related to others. The first dimension of materialism correlates with face and fully mediates the relationship between face and online purchases, whereas the second does not, further showing that this dimension of materialism is socially directed. Moreover, both of the dimensions influence online consumption, while the impact of the first dimension is much stronger than that of the second, as social influence greatly drives consumption in China. This result verifies He et al.’s (2018) finding that the main motivation for online buying among Chinese consumers is “observed buying” rather than “unobserved buying” which is more common in Western societies.

In addition, there is no direct correlation between price consciousness and online consumption. This may be the case because price consciousness has converse effects on online purchases. First, price-conscious consumers are likely to be frugal (Shoham and Brencic, 2004), and they thus will restrict their desire for material goods. The correlation matrix shows that price consciousness negatively relates to the acquisition centrality dimension of materialism. In another respect, inexpensive products sold one-commerce platforms may greatly attract price-conscious consumers, leading to a greater online purchasing tendency. The two opposing forces counteract each other. However, price consciousness is a catalyst that strengthens the relationship between the combined dimension of success and happiness for materialism and online consumption. This result demonstrates that highly price-conscious consumers are more likely to make online purchases to achieve gratification by exhibiting possession-defined success.

## Limitation and Future Research

There is no study without limitations. First, we only use a general item to measure consumers’ online purchase frequency in this study. Future research should develop multidimensional scales that measure consumers’ buying behavior across different categories of online products. Such work would help brand managers develop appropriate strategies for this large market.

In addition, this study focuses on the younger generation between 20 and 30 years of age because this cohort makes the majority of online purchases. Considering that e-commerce is so prevalent across China, it will be necessary to extend the present research to other generation cohorts. Moreover, the current study was conducted online. We did not consider the regions/cities where the respondents lived even though within-country diversity in consumer preferences and behavior has been found in China (Zhang et al., 2008). Future research should extend the present study to drawn cross-regional comparisons.

Finally, in our contemporary consumption-oriented society where materialism is a sign of prosperity (Borgmann, 2000), the materialism construct is attracting the attention a great number of consumer researchers. However, the classic three-factor structure of Richins and Dawson’s (1992) materialism scale often fails to validate expectations for non-English-speaking countries. For instance, in assessing the scale in Denmark, France, and Russia, Griffin et al. (2004) found the scale to display an adequate fit to the Danish sample but to be poorly suited to their French and Russian samples. Moreover, the factor structures found in China, France, and Russia differ from each other. Thus, there is an urgent need to measure materialism in cross-cultural contexts and explore its characteristics in each society, which will greatly benefit future marketing research.

## Data Availability Statement

The raw data supporting the conclusions of this article will be made available by the authors, without undue reservation.

## Ethics Statement

Ethical review and approval was not required for the study on human participants in accordance with the local legislation and institutional requirements. Written informed consent for participation was not required for this study in accordance with the national legislation and the institutional requirements.

## Author Contributions

GS developed the theoretical framework and worked on literature review and manuscript writing. FS was in charge of data collection and analysis. XM took part in literature review and data analysis. All authors contributed to the article and approved the submitted version.

## Conflict of Interest

The authors declare that the research was conducted in the absence of any commercial or financial relationships that could be construed as a potential conflict of interest.

## Publisher’s Note

All claims expressed in this article are solely those of the authors and do not necessarily represent those of their affiliated organizations, or those of the publisher, the editors and the reviewers. Any product that may be evaluated in this article, or claim that may be made by its manufacturer, is not guaranteed or endorsed by the publisher.

## Footnotes

1
http://www.sojump.com
